# Fungal GH25 muramidases: New family members with applications in animal nutrition and a crystal structure at 0.78Å resolution

**DOI:** 10.1371/journal.pone.0248190

**Published:** 2021-03-12

**Authors:** Olga V. Moroz, Elena Blagova, Edward Taylor, Johan P. Turkenburg, Lars K. Skov, Garry P. Gippert, Kirk M. Schnorr, Li Ming, Liu Ye, Mikkel Klausen, Marianne T. Cohn, Esben G. W. Schmidt, Søren Nymand-Grarup, Gideon J. Davies, Keith S. Wilson

**Affiliations:** 1 Department of Chemistry, Structural Biology Laboratory, The University of York, York, United Kingdom; 2 Novozymes A/S, Kongens Lyngby, Denmark; 3 Novozymes Investment Co. Ltd., Beijing, China; Weizmann Institute of Science, ISRAEL

## Abstract

Muramidases/lysozymes hydrolyse the peptidoglycan component of the bacterial cell wall. They are found in many of the glycoside hydrolase (GH) families. Family GH25 contains muramidases/lysozymes, known as CH type lysozymes, as they were initially discovered in the *Chalaropsis* species of fungus. The characterized enzymes from GH25 exhibit both β-1,4-*N*-acetyl- and β-1,4-*N*,6-O-diacetylmuramidase activities, cleaving the β-1,4-glycosidic bond between *N*-acetylmuramic acid (NAM) and *N*-acetylglucosamine (NAG) moieties in the carbohydrate backbone of bacterial peptidoglycan. Here, a set of fungal GH25 muramidases were identified from a sequence search, cloned and expressed and screened for their ability to digest bacterial peptidoglycan, to be used in a commercial application in chicken feed. The screen identified the enzyme from *Acremonium alcalophilum* JCM 736 as a suitable candidate for this purpose and its relevant biochemical and biophysical and properties are described. We report the crystal structure of the *A*. *alcalophilum* enzyme at atomic, 0.78 Å resolution, together with that of its homologue from *Trichobolus zukalii* at 1.4 Å, and compare these with the structures of homologues. GH25 enzymes offer a new solution in animal feed applications such as for processing bacterial debris in the animal gut.

## 1. Introduction

Muramidases (*N*-acetylmuramide glycanhydrolases), also known as lysozymes, cleave the bacterial cell wall peptidoglycan component at the β-1,4-glycosidic bond between *N*-acetylmuramic acid (NAM) and *N*-acetylglucosamine (NAG) in the carbohydrate backbone. They were first discovered in 1922 by Fleming, who observed antibacterial action when he treated bacterial cultures with nasal mucus from a patient suffering from a cold; he gave the enzyme the name “lysozyme’ [1] and showed that similar enzymes were found in a number of other animals, including hens (hen egg white lysozyme, HEWL). These enzymes were subsequently classified in glycoside hydrolase (GH) family 22 (see the Carbohydrate Active Enzymes database (CAZy; http://www.cazy.org/) [2,3]. The Enzyme Commission assigned these proteins the number E.C.3.2.1.17 and recommended that the name lysozyme be replaced by muramidase or *N-*acetylmuramide glycanohydrolase [4]. However, the name lysozyme has persisted, together with several alternatives, including 1,4-*N*-acetylmuramidase mucopeptide *N*-acetylmuramoylhydrolase; PR1-lysozyme—see a full list in the CAZy database, as well as lysins (mostly for phage enzymes). The name muramidase will be used throughout the present report.

Muramidases are found in several GH families annotated in CAZy: GH22, GH23, GH24, GH25, GH73, GH104 and GH108. Although all these families contain members that cleave the same substrate, they do so by a number of different mechanisms. The focus of the present study is on the fungal members of the GH25 family: (https://www.cazypedia.org/index.php/Glycoside_Hydrolase_Family_25), also called the *Chalaropsis* (CH) type, based on their initial characterisation in this species of fungus [5,6] and from the first structure of a family member, the bacterial *Streptomyces coelicolor* [7]. GH25 muramidases are mainly present in microorganisms, with diverse functions from remodelling of the cell wall in bacteria during cell division [8] to lysing the bacterial cell wall at the end of the phage life cycle in bacteriophage, and antibacterial action in archaea and eukaryotes [9]. In the latter comparative genomics study, it was suggested that the ancestral gene originated from bacteria and spread through other domains of life by horizontal gene transfer, where non-bacterial organisms used it to compete in a bacterial world. Many of the fungal muramidases are clearly extracellular since they include a signal secretion peptide [10].

Muramidases from GH22 are used in a number of industrial applications, such as antibacterials for food conservation, as an additive to infant milk formula, in the cheese industry [11] or in biomedical research for facilitating degradation of bacterial cell walls during protein expression in *Escherichia coli* ([12] and references therein). Our aim was to identify a different type of muramidase to act as a feed additive for processing bacterial debris in the gut of animals (particularly chickens). For this application gastric stability is an important property that limits the use of HEWL as a feed additive [13]. We further required that the enzyme is not toxic to the usual set of bacterial flora—i.e. will not disrupt normal healthy bacterial activity in the gut. Hence, in the initial screening we searched for a gastric stable muramidase with a broad promiscuous specificity on peptidoglycan, which was not too active and would probably have low “normal” antibacterial activity. Other parameters, such as temperature stability, high expression levels in our normal production hosts, and *in vivo* testing, were also important. A total of 55 fungal GH25s were screened, substantially increasing the number of characterized fungal GH25s.

The enzyme from *Acremonium alcalophilum *JCM 7366 (*A*. *alcalophilum* is now known as *Sodiomyces alcalophilus* (G. Okada) Giraldo López & Crous, but we will use the *Aa*Mur abbreviation for the muramidase in this paper) finally proved to be the muramidase of choice. *A*. *alcalophilum* was first isolated from pig manure over 30 years ago by a Japanese researcher, A. Yoneda, and provides a genetic resource for identifying extracellular enzymes with pH optima in the alkaline range [14]. *Aa*Mur was originally included in the diversity generation because of this interesting alkaline property, but as described later it has optimum in the slightly acidic range. Later *Aa*Mur was expressed in a *Trichoderma reesei* production host, evaluated as a feed additive in a toxicology study [15] and recently developed into a commercial product [16,17] (Balancius^TM^ from DSM nutritional products, Kaiseraugst, Switzerland) that gives improved utilization of feed ingredients. It is believed that the mode of action for *Aa*Mur in the feed application is to break down cell debris peptidoglycans in the gut, which would otherwise be blocking the villi, thereby increasing nutrient uptake. It was demonstrated that the product is safe in use for chickens and for consumers and that, with the dose used in the application, *Aa*Mur did not have an antimicrobial effect on the bacteria tested [18], including *E*. *coli* ATCC 25922, *Pseudomonas aeruginosa* ATCC 27853, *Staphylococcus aureus* ATCC 25923, *Enterococcus faecalis* ATCC 29212, *Bacillus subtilis* ATCC 6633, *Campylobacter jejuni* ATCC 33560 and *Clostridium perfringens* ATCC 13124. In a recent study it was shown that the plate count results for *Lactobacillus* and enterobacteria did not show a significant difference with the inclusion of the muramidase in chicken feed when looking at samples from crop, ileum or cecum [19]. As stated in the previous paragraph these were the low antimicrobial properties we were looking for in the initial screening.

GH25 muramidases are structurally unrelated to the other lysozyme families and have a modified α/β-barrel-like fold which differs from the classical (βα)_8_ “TIM-barrel” (where TIM stands for triose phosphate isomerase [20,21]) in that its eight-stranded β-barrel is only flanked by five α-helices, rather than eight, so is called (βα)_5_ (β)3—first described for the *Streptomyces coelicolor* enzyme “cellosyl” (1JFX [7]). There are structures in the PDB for three bacteriophage, six bacterial but only one fungal GH25 enzyme, Table 1. The structure of modular autolysin LytC from *Streptococcus pneumoniae* was in addition obtained in complex with a pneumococcal peptidoglycan domain. The enzyme from *Clostridium perfringens* phage phiSM101 is of particular interest because it is a complex with the ligand, 2-(acetylamino)-2-deoxy-α-D-glucopyranose (NDG), located in the binding site close to the active motif DXE, discussed in Results and discussion section.

**Table 1:** Structures of family GH25 muramidases in the PDB.

Enzyme	Species	PDB	Complex	Reference
Endolysin PlyB	Bacteriophage P40 of *Listeria monocytogenes*	4JZ5		None.
Clp-1 lysozyme	Bacteriophage of *Streptococcus pneumoniae*	1H09, 1OBA, 2IXU, 2J8G, 2J8F, 2IXV		[22,23]
Muramidase	*Clostridium perfringens* phage phiSM101	4KRT, 4KRU	2-(acetylamino)-2-deoxy-a-d-glucopyranose (NDG)	[24]
Muramidase	*Bacillus anthracis* Ames	2WAG		[25])
Muramidase	*Bacillus anthracis* Sterne	3HMC		[26]
S40 SleM CLE muramidase	*Clostridium perfringens*	5JIP		[27]
Modular autolysin LytC	*Streptococcus pneumoniae* R6	2WWD, 2WWC, 2WW5	Pneumococcal peptidoglycan fragment	[28]
Virulence factor	*Streptococcus pneumoniae* TIGR4	4FF5		[29]
Muramidase	*Streptomyces coelicolor*	1JFX		[7]
Muramidase	*Aspergillus fumigatus*	2X8R		[30]

Here we report on the selection of *Aa*Mur after screening a set of fungal muramidases. Selection criteria included activity against peptidoglycan from *Micrococcus lysodeikticus* and *Lactobacillus johnsonii*, tolerance to the environment in a poultry gut and pH profile. In addition, we present the crystal structures of *Aa*Mur and also of the homologous *Trichobolus zukalii* (henceforth named *Tz*Mur). The structures are compared with those of previously determined GH25 enzymes.

## 2. Materials and methods

### 2.1 Screening for a suitable fungal muramidase

55 fungal GH25 muramidases were identified by bioinformatic tools exploiting public and internal Novozymes strain collections and databases, and were subsequently cloned and expressed by standard methods [31]. Unless otherwise stated all chemicals/reagents were purchased from Sigma-Aldrich and were reagent grade. Purifications were carried out by standard techniques, typically involving cation or anion exchange. Details about the cloning and expression of *Aa*Mur and *Tz*Mur, and the purification of *Aa*Mur can be found in the Supporting information. Muramidase activity was measured for the 55 enzymes using the turbidity and the reducing ends assays detailed below. The turbidity assay was also used in the test for gastric stability.

#### 2.1.1. Preparation of peptidoglycan for assays

Lyophilized cells of *Micrococcus lysodeikticus* ATTC No. 4698 were obtained from Sigma-Aldrich (M3770) and were used as the peptidoglycan source in the various assays described below. Peptidoglycan was extracted from a cultivation of *Lactobacillus johnsonii* DSM10533 (see procedure in Supporting information).

#### 2.1.2. Activity assay by reduction in turbidity (the OD-drop assay)

The OD-drop assay measures muramidase/lysozyme activity through a reduction in optical density (OD) caused by turbidity (light scattering) as described in many papers on HEWL [32,33]. Enzyme activities at 37°C were determined by measuring the decrease (drop) in optical density of a solution of resuspended *M*. *lysodeikticus* ATTC No. 4698 (Sigma-Aldrich M3770) measured in a spectrophotometer at 450 nm ([34] and https://www.sigmaaldrich.com/technical-documents/protocols/biology/enzymatic-assay-of-lysozyme.html). Before use, *M*. *lysodeikticus* cells were resuspended in citric acid/phosphate buffer pH 4.0 to a concentration of 0.5 mg cells/mL and the OD at 450 nm was measured. The cell suspension was adjusted so the cell concentration equaled an OD_450_ of 1.0 and the adjusted cell suspension was stored at 4°C before use. Resuspended cells were used within four hours. The values in Table 4 are averages of three determinations of the reductions in OD_450_ after 60 minutes reaction time with a muramidase concentration of 4.5 μg/mL

#### 2.1.3 Gastric stability

The muramidases were challenged by a 15 minute incubation at pH 3.0 and 37°C in the presence of pepsin (474 U/mL) before the residual activity toward *M*. *lysodeikticus* was measured using the OD-drop method described above. The muramidases were dosed as 4.5 μg/mL in the assay and the activity of the muramidases was determined at pH 4.0. Activity of the challenged sample was compared to an unchallenged sample and the resulting residual activity (in %) recorded.

#### 2.1.4 Activity on peptidoglycan at pH 5.0 using a reducing ends assay

When peptidoglycan is hydrolysed by a muramidase new saccharide reducing ends (aldehyde groups) are produced and the increase in reducing ends is used as a measure of glycolytic activity. After incubation, the amount of reducing ends produced was determined by reaction with para-hydroxybenzoic acid hydrazide. The resulting hydrazone has a yellow colour and can be detected at 405 nm.

The muramidases were diluted in phosphate dilution buffer (5 mM citrate, 5 mM K_2_HPO_4_, 0.01% TritonX-100, pH 5.0) to 200 or 50 μg/mL in polypropylene tubes, dependent on the strength of available stock solutions. The solutions were further diluted in a 96-well polypropylene microtiter plate by preparing a two-fold dilution series down to a concentration of 6.3 μg/mL in phosphate dilution buffer. The incubation can be performed at different pHs (see section 2.2.4), here we used pH 5.0 in order to be closer to intestinal pH. The assay can be performed with peptidoglycan from several source, we describe it below with *L*. *johnsonii* as an example.

A 50 mg/mL stock solution of *L*. *johnsonii* substrate in water was prepared and diluted in phosphate buffer (50 mM citrate, 50 mM K_2_HPO_4_, pH 5.0) to 250 μg/mL. In a polypropylene deep-well plate 50 μL of the muramidase dilution was mixed with 450 μL *L*. *johnsonii* solution and incubated at 40°C with shaking (500 rpm) for 45 min. After incubation, the deep-well plates were centrifuged (3200 rpm, 7 min) to pellet insoluble material and 100 μL of the supernatant was mixed with 50 μL 3.2 M HCl in a 96-well PCR plate and incubated at 95°C for 80 min. 50 μL of 3.5 M NaOH was added to each well of the PCR plate, and 150 μL of each sample was transferred to a new PCR plate containing 75 μL/well 4-hydroxybenzhydrazide (PAHBAH) solution in K-Na tartrate/NaOH buffer (50 g/L K-Na tartrate + 20 g/L NaOH). The plate was incubated at 95°C for 10 min before 100 μL/sample was transferred to a clear flat-bottomed microtiter plate for optical density (OD) measurement at 405 nm. OD measurements were performed on three times diluted samples (50 μL sample diluted in 100 μL in Milli-Q water at 25°C). The OD measurement values shown in Table 4 represent the difference after the original (background) reading was subtracted and are the average of two OD measurement values.

### 2.2 Further investigations of AaMur and TzMur

Following the screening of the fungal enzymes, biochemical and biophysical characterisation was performed on selected muramidases, primarily *Aa*Mur and *Tz*Mur.

#### 2.2.1 Thermostability using nano Differential Scanning Fluorescence

Nano Differential Scanning Fluorescence (nanoDSF) was performed on eight of the muramidases with a Prometheus NT.48 instrument (NanoTemper Technologies GmbH, München, Germany). Purified samples (in either 50 mM Na-acetate, pH 3.0, or 50 mM Na-acetate, pH 5.0) were loaded into nanoDSF standard grade capillaries (NanoTemper Technologies GmbH) through capillary action. Three capillaries were filled for each sample, placed into the instrument (up to 48 single capillaries can be loaded in a single run) and the laser intensity required for optimum signal generation was determined. The samples were run with the following experimental setting: temperature slope 2°C/minute, start temperature 20°C and end temperature 95°C. The data were analysed using the software supplied with the instrument (PR.ThermControl v2.0.4, NanoTemper Technologies GmbH) and the melting point, T_m_ (°C), (for the ratio 350 nm/330 nm) was determined.

#### 2.2.2 Thermostability of *Aa*Mur at pH 4.5, 5.5 and 6.5 using Differential Scanning Calorimetry

An aliquot of purified *Aa*Mur was buffer-exchanged (50 mM Na-acetate, pH 4.5; 50 mM Na-acetate, pH 5.5; or 50 mM MES (2-(N-morpholino)ethanesulfonic acid), pH 6.5) using prepacked PD-10 columns. The sample was 0.45 μm filtered and diluted with buffer to approximately 2 A_280_ units, with the buffer as reference solution. The thermostability of the muramidase at different pH values was determined by Differential Scanning Calorimetry (DSC) using a VP-capillary DSC instrument (MicroCal Inc., Piscataway, NJ, USA) equipped with an auto sampler. The thermal melting point, T_m_, was taken as the top of the denaturation peak (major endothermic peak) in the thermograms (Cp vs. T) obtained after heating the muramidase solutions in the buffer at a constant programmed heating rate. Sample and reference solutions (approx. 0.5 mL) were thermally pre-equilibrated for 10 minutes at 20°C and the scan was performed from 20 to 110°C at a scan rate of 200 K/hour. Data-handling was performed using the MicroCal Origin software (version 7.0383). T_m_’s were determined with an accuracy of approximately +/- 0.5°C.

#### 2.2.3 pH optimum using the OD assay

The pH activity profiles of *Aa*Mur and *Tz*Mur on *M*. *lysodeikticus* peptidoglycan were determined using a modified OD-drop assay. The *M*. *lysodeikticus* substrate was prepared as described under “Activity assay by reduction in turbidity (the OD-drop assay)” except that the citric acid–phosphate buffer was adjusted to one of the following pH values with HCl or NaOH prior to the start of the experiment: 3.0, 3.5, 4.0, 4.5, 5.0, 5.5, 6.0, 6.5 and 7.0.

#### 2.2.4 pH-profiles of *Aa*Mur and *Tz*Mur using the reducing end assay

The reducing end assay was run at a range of pH in the incubation step (Buffer: 100 mM Glycine, 100 mM KH_2_PO_4_, 100 mM MES, 0.01% TX-100, adjusted to appropriate pH 3–10) using *M*. *lysodeikticus* as substrate.

### 2.3. Structure solution for *Aa*Mur and *Tz*Mur

For both proteins, the initial crystallisation was carried out in a number of commercial screens using sitting-drop vapour-diffusion with drops set up using a *Mosquito Crystal* liquid handling robot (SPT LabTech, UK) with 150 nL protein solution plus 150 nL reservoir solution in 96-well format plates (MRC 2-well crystallization microplate, Swissci, Switzerland) equilibrated against 54 μL reservoir solution. All computations were carried out using programs from the CCP4 suite [35], unless otherwise stated. Data collection and processing and final refinement statistics are given in Table 2. Structure figures were drawn with CCP4mg [36]. The quality of the final models was validated using Molprobity [37].

**Table 2:** Crystallographic statistics. Values in parentheses are for the outer resolution shell.

	*Aa*Mur	*Tz*Mur
Diffraction source	Diamond I24	Diamond I04
Wavelength (Å)	0.65	0.98
Space group	P2_1_	P1
*a*, *b*, *c* (Å)	34.087, 77.272, 35.728	39.398, 48.582, 55.737
α, β, γ (°)	90.0, 104.2, 90.0	97.1, 99.5, 98.2
Resolution range (Å)	38.64–0.78 (0.79–0.78)	27.17–1.40 (1.42–1.40)
Total No. of reflections	793,527 (24,210)	155,691 (5146)
No. of unique reflections	201,033 (8,829)	73,721 (2699)
Completeness (%)	99.1 (87.6)	93.8 (69.0)
Redundancy	3.9 (2.7)	2.1 (1.9)
CC(1/2)^a^^)^	0.979 (0.356)	0.992 (0.956)
〈*I*/σ(*I*)〉	5.4 (0.8)	15.8 (7.6)
*R*_*merge*_ ^b^^*)*^	0.149 (1.456)	0.045 (0.122)
*R*_r.i.m._^c^^)^	0.082 (0.996)	0.038 (0.103)
Overall *B* factor: Wilson plot (Å^2^)	5.4	5.05
Refinement		
No. reflections, working set	200,983	73721
No. reflections, test set	10,021	3717
Final *R*_cryst_ (%)	11.6	9.7
Final *R*_free_ (%)	13.0	13.7
No. of non-H atoms		
Protein	1,768	3,353
Solute	12	85
Water	331	690
Total	2,111	4,128
R.m.s. deviations		
Bonds (Å)	0.0216	0.0175
Angles (°)	1.83	1.94
Average *B* factors (Å^2^)		
Protein	8.8	8.4
Solute	26.1	18.6
Water	23.7	34.7
Molprobity score	1.62	1.48
Ramachandran plot		
Most favoured (%)	98.1	98.3
Outliers (%)	0.0	0.0
PDB code	6ZM8	6ZMV

a) CC_1/2_ values for Imean are calculated by splitting the data randomly in half.

b) R_merge_ is defined as Σ_hkl_Σ_j_|I_hk,j_—<I_hk_>|/Σ_hkl_Σ_j_ I_hk,j_ where I is the intensity of the reflection.

c) Redundancy-independent merging R factor R_r.i.m_ [38].

#### 2.3.1. Acremonium alcalophilum

Crystals grew in multiple conditions of the initial screens. Data were collected from several crystals cryoprotected with 30% ethylene glycol (3μl ethylene glycol added to 6μl of well solution) and the best dataset, from JCSG C1 (0.2 M NaCl, 0.1 M phosphate/citrate pH 4.2, 20% w/v PEG 8K) was selected for refinement. Data were collected at the Diamond Light Source, beamline I24 to a resolution of 0.78 Å, and processed using XDS [39] within the xia2 pipeline [40], and merged in Aimless [41]. The structure was solved by molecular replacement with MOLREP [42], using as search model the GH25 enzyme from *Aspergillus fumigatus* (PDB code 2X8R [30]). The structure was rebuilt with Buccaneer and refined with alternating cycles of REFMAC5 [43] and COOT [44].

#### 2.3.2. Trichobolus zukalii

A number of clusters of crystals grew in the first Ammonium Sulphate screen (Qiagen). Due to the limited amount of material, the drops were re-dissolved to obtain a sample for manual optimisation, with the final crystals obtained in a 24 well ’Linbro-style’ vapour diffusion crystal growth plate, in a hanging drop set up. The final conditions were 0.6–0.7 M lithium-sulphate, 0.5 M ammonium sulphate, 0.1 M Na-citrate. These were mostly inter-grown plates from which a number were separated and tested in house, where they diffracted to around 2.2 Å. The crystals were cryo-protected with 25% glycerol added to the crystallisation medium. Data were collected at the Diamond Light Source beamline I04-1 to 1.4Å resolution and integrated XDS within xia2 [40]. The structure was solved by molecular replacement using Molrep with the GH25 enzyme from *Aspergillus fumigatus*, PDB code 2X8R as search model. The chain was rebuilt manually, and the structure refined with alternating cycles of REFMАC and COOT.

## 3. Results and discussion

### 3.1. Screening for an optimal fungal GH25 muramidase

**Fig 1:**
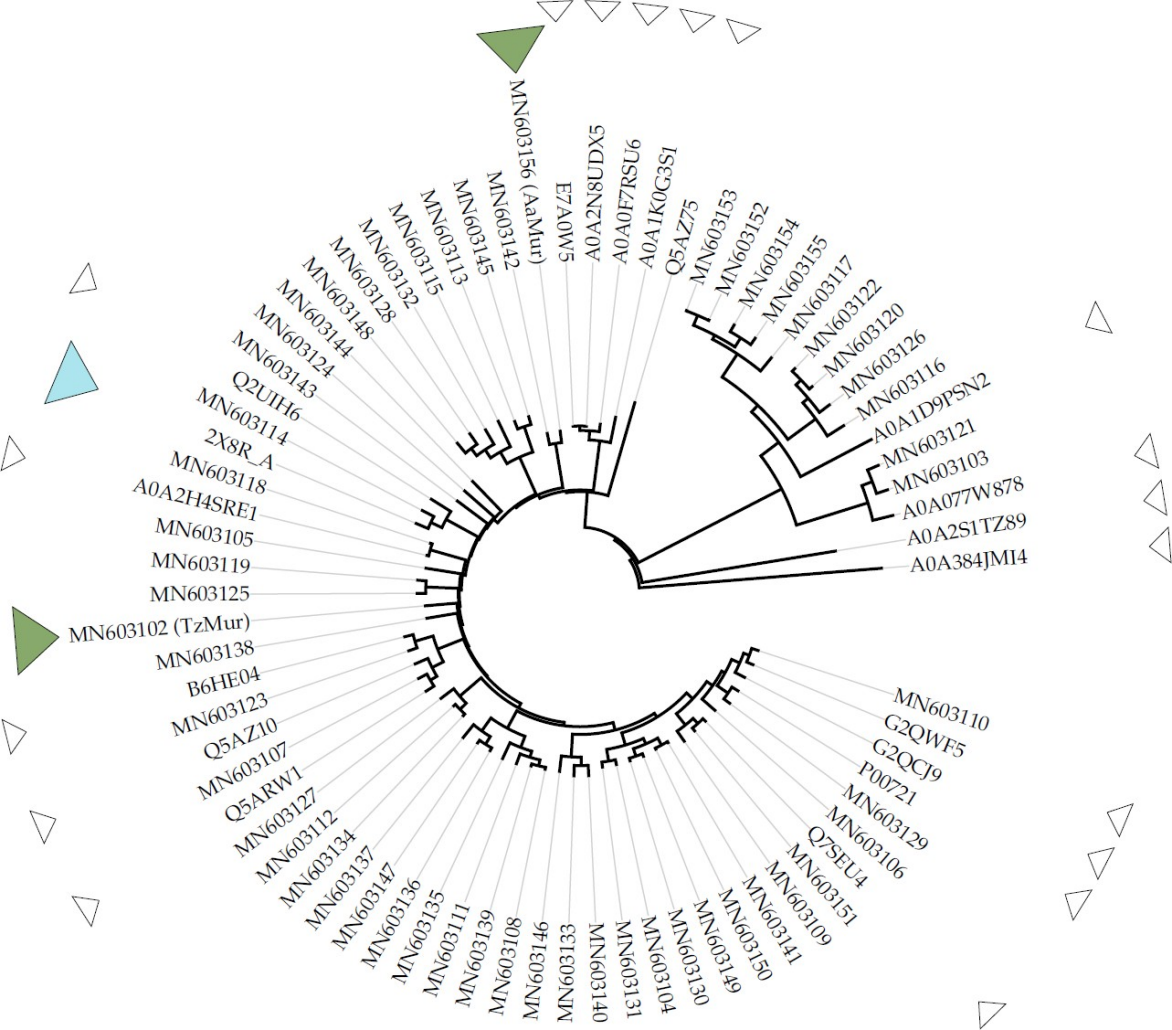
Evolutionary tree of fungal GH25 muramidases. The set of 55 GH25 sequences included in the screening process (GenBank accession id:MN603101 to MN603156) and public fungal GH25 sequences in the CAZy-database [3] (https://www.cazypedia.org/index.php/Glycoside_Hydrolase_Family_25). The open triangles indicate a public sequence, the light-blue triangle is a fungal GH25 structure from the PDB while the green triangles indicate the structures reported here. The organism names of MN603101-MN603156 are given in Table 3.

Genes for the set of fungal GH25 muramidases were cloned and the corresponding enzymes overexpressed using standard procedures as described in Materials and Methods and in the Supporting information. The evolutionary tree of the complete set of 55 GH25s and public fungal GH25 sequences in the CAZy-database is shown in Fig 1. There is presently a total of over 10,000 sequences for GH25 family enzymes in the database, but the vast majority are of bacterial origin. Only 49 are from eukaryotes, so the 55 muramidases presented here is a significant expansion of the fungal GH25 diversity. Out of the 10,000 sequences listed in the database only 31 are listed as “characterized”, so there is a huge lack of biochemical data on GH25 muramidases.

**Table 3:** The screening results for the 55 fungal muramidases.

NCBI ID	Organism	OD on Ml pH 4.0	% Gastric stability	RE Ml	RE Lj
MN603102	*Trichobolus zukalii* (*Tz*Mur)	0.10	99	1.98	0.26
MN603103	*Rhizomucor pusillus*	0.17	127	1.34	0.01
MN603104	*Poronia punctata*	0.19	28	1.78	0.14
MN603105	*Poronia punctata*	0.14	-2	0.85	0.01
MN603106	*Zopfiella sp*.	0.29	34	2.22	0.24
MN603107	*Aspergillus deflectus*	0.25	84	0.99	0.02
MN603108	*Pycnidiophora sp*.	0.22	108	2.32	0.1
MN603109	*Cladorrhinum bulbillosum*	0.27	35	2.14	0
MN603110	*Arcopilus cupreus*	0.14	103	1.71	0.19
MN603111	*Sporormia fimetaria*	0.22	88	2.60	0.18
MN603112	*Paecilomyces sp*.	0.26	40	2.06	0.14
MN603113	*Paecilomyces sp*.	0.26	17	1.98	0.12
MN603114	*Aspergillus sp*.	0.22	20	0.93	0.19
MN603115	*Onygena equina*	0.24	ND	1.76	0.16
MN603116	*Penicillium citrinum*	0.14	ND	1.20	-0.02
MN603117	*Deconica coprophila*	0.17	101	1.80	-0.07
MN603118	*Lecanicillium sp*.	0.17	ND	1.79	0.4
MN603119	*Lecanicillium sp*.	0.24	55	2.03	0.17
MN603120	*Cordyceps farinosa*	0.16	100	1.72	-0.03
MN603121	*Thermomucor indicae-seudaticae*	0.21	36	1.38	-0.05
MN603122	*Lecanicillium sp*.	0.08	55	1.47	-0.08
MN603123	*Penicillium sp*. *qii*	0.29	97	2.07	0.27
MN603124	*Malbranchea flava*	0.24	0	1.56	0.34
MN603125	*Parengyodontium album*	0.24	150	2.40	0.59
MN603126	*Blackwellomyces cardinalis*	0.14	ND	1.94	0
MN603127	*Purpureocillium lilacinum*	0.24	135	2.88	0.12
MN603128	*Umbelopsis westeae*	0.19	104	2.33	0.24
MN603129	*Coniochaeta sp*.	0.22	88	2.11	0.07
MN603130	*Daldinia fissa*	0.17	30	1.30	0.04
MN603131	*Rosellinia sp*.	0.18	115	1.59	0.24
MN603132	*Flammulina velutipes KACC42780*	0.26	83	1.74	0.17
MN603133	*Hamigera paravellanea*	0.25	ND	1.74	0.02
MN603134	*Metapochonia bulbillosa*	0.25	93	2.09	0.18
MN603135	*Curreya sp*.	0.28	46	1.91	0.13
MN603136	*Ascobolus sp*.	0.29	108	1.79	0.12
MN603137	*Coniothyrium sp*.	0.29	51	1.23	0.12
MN603138	*Yunnania penicillata*	0.15	57	1.28	0.14
MN603139	*Westerdykella sp*.	0.15	90	1.94	0.19
MN603140	*Paracremonium inflatum*	0.11	133	2.59	0.08
MN603141	*Xylariaceae sp*.	0.16	39	1.52	0.05
MN603142	*Clonostachys rossmaniae*	0.11	86	1.24	0.02
MN603143	*Aspergillus inflatus*	0.07	88	2.03	-0.02
MN603144	*Metarhizium sp*.	0.03	95	2.24	-0.02
MN603145	*Simplicillium obclavatum*	0.15	78	1.68	0.21
MN603146	*Thermoascus aurantiacus*	0.09	61	2.39	0.15
MN603147	*Ascobolus sp*.	0.23	82	1.34	-0.01
MN603148	*Zygomycetes sp*.	0.21	98	2.52	0.69
MN603149	*Hypoxylon sp*.	0.25	113	1.50	0.28
MN603150	*Hypoxylon sp*.	0.28	131	1.77	0.05
MN603151	*Gelasinospora cratophora*	ND	111	1.65	-0.09
MN603152	*Stropharia semiglobata*	ND	47	1.34	0.25
MN603153	*Stropharia semiglobata*	ND	104	1.52	0.16
MN603154	*Stropharia semiglobata*	ND	105	1.01	0.29
MN603155	*Hypholoma polytrichi*	ND	130	1.86	0.57
MN603156	*Acremonium alcalophilum JCM 7366* (*Aa*Mur)	0.18	103	0.52	0.49

**Fig 2:**
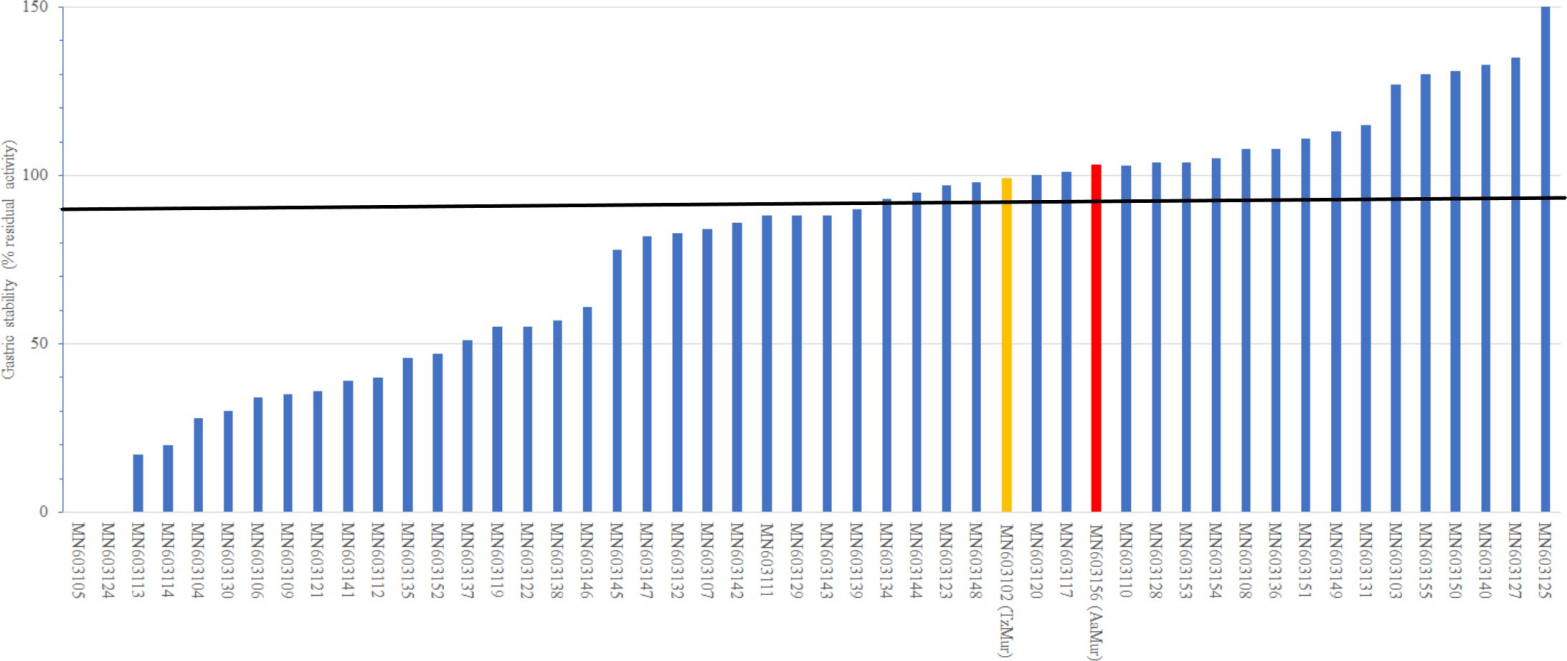
Gastric stability (% residual activity using the OD-drop assay) of the screened muramidases. *Aa*Mur is coloured red and *Tz*Mur is coloured yellow. The horizontal line is at 90% survival.

After purification the enzymes were screened by the OD-drop assay, gastric stability, and the reducing end assay (on two peptidoglycan sources). The OD-drop assay measures muramidase/lysozyme activity through a reduction in optical density (OD) caused by turbidity (light scattering), while the reducing ends assay determines the solubilisation of carbohydrates from peptidoglycan. The OD-drop assay was meant to check for muramidase activity, but it was found that a low value in this assay does not exclude muramidase activity on other types of peptidoglycans. Gastric stability measured with the OD-drop assay was one of the key selection parameters since the muramidase needs to survive the stomach before it can work in the intestines. The simulated gastric stability of the 55 muramidases at pH 3 in the presence of pepsin showed that several (including *Aa*Mur and *Tz*Mur) were gastric stable (Table 3 and Fig 2). The aim was to identify an enzyme with high gastric stability, and candidates with values above ~90% were considered to be sufficiently stable. Due to the high variation in the OD-drop assay some residual activities were above 100%, however these were not considered more stable. All the screening data are summarised in Table 3.

The columns are as follows: 1) sequence identifier (NCBI code), 2) species name (some organisms have more than one GH25), 3) Activity on *M*. *lysodeikticus* using the OD-drop assay at pH 4.0 and 4.5 μg/mL, 4) Gastric stability in %-survival after incubation at pH 3.0 in the presence of pepsin, 5) Activity (OD change at 405 nm) on *M*. *lysodeikticus* using the reducing ends (RE) assay at pH 5.0 and 0.7 μg/mL, 6) Activity (OD change at 405 nm) on *L*. *johnsonii* peptidoglycan pH 5.0 at 1.25 μg/mL.

*M*. *lysodeikticus* is an obligate aerobe that is found on mammalian skin, in the mouth and in the upper respiratory tract while *L*. *johnsonii* is an aerotolerant anaerobes. *L*. *johnsonii* is a representative of Lactobacilli that is part of the gut microbiota [45]. In the screening *L*. *johnsonii* was therefore considered more application relevant than *M*. *lysodeikticus*. It can be seen from Table 3 that 10 muramidase were gastric stable (>90% residual activity) and had values of more than 0.2 in the reducing ends assay on peptidoglycan from *L*. *johnsonii*.

### 3.2. Temperature stability and pH optimum/profiles of AaMur and TzMur

#### 3.2.1. Temperature stability by nano-DSF for *Aa*Mur, *Tz*Mur and 6 other GH25s

The melting temperatures, T_m_, were determined using nano-DSF for 8 of the GH25 muramidases at pH 3.0 and 5.0 (Table 4). Nano-DSF was not available to us during the initial screening of the 55 enzymes. Most material was used for other experiments, so nano-DSF was only carried out on candidates where sufficient material was available (reflecting expressibility in the fungal host *Aspergillus oryzae*).

**Table 4:** The melting points of a subset of the GH25 enzymes determined by nano-differential scanning fluorimetry.

Organism	NCBI ID	T_m_ (°C)	T_m_ (°C)
		**pH 3.0**	**pH 5.0**
*Trichobolus zukalii (Tz*Mur*)*	MN603102	63.9	66.6
*Rhizomucor pusillus*	MN603103	76.0	73.9
*Poronia punctata*	MN603104	55.7	53.8
*Poronia punctata*	MN603105	56.2	54.5
*Zopfiella sp*. *t180-6*	MN603106	70.2	62.9
*Aspergillus deflectus*	MN603107	52.5	64.2
*Ascobolus stictoideus*	MN603147	64.1	64.2
*Acremonium alcalophilum (Aa*Mur*)*	MN603156	69.5	68.1

From these experiments, the enzymes from *Rhizomucor pusillus* and *Acremonium alcalophilum* appeared to be the best candidates with the highest T_m_ (68°C and above) at the two pH. However, *Rhizomucor pusillus* (MN603103) had low activity on peptidoglycan from *L*. *johnsonii* (Table 3) and was not selected. *Tz*Mur, *Zopfiella sp*. *t180-6* and *Ascobolus stictoideus* had T_m_ above 60°C at both pH 3 and 5. The melting temperature of *Aa*Mur as a function of pH using three different buffers is shown in Table 5, establishing that it retained its stability over a range of pH.

**Table 5:** Melting temperatures of *Aa*Mur by DSC in three different buffers.

Buffer	T_m_ (°C)
50 mM Na-acetate, pH 4.5	71.2
50 mM Na-acetate, pH 5.5	67.3
50 mM MES (2-(*N*-morpholino)ethanesulfonic acid), pH 6.5	65.8

On the basis of the screening assays (section 2.1), thermostability at pH 3–5 (section 2.2.1), expressibility in a suitable host and general manufacturability, the enzymes from *Acremonium alcalophilum* and *Trichobolus zukalii*, *Aa*Mur and *Tz*Mur, were selected as having appropriate properties and were actively pursued for further investigation.

#### 3.2.2. Activity as a function of pH

**Fig 3:**
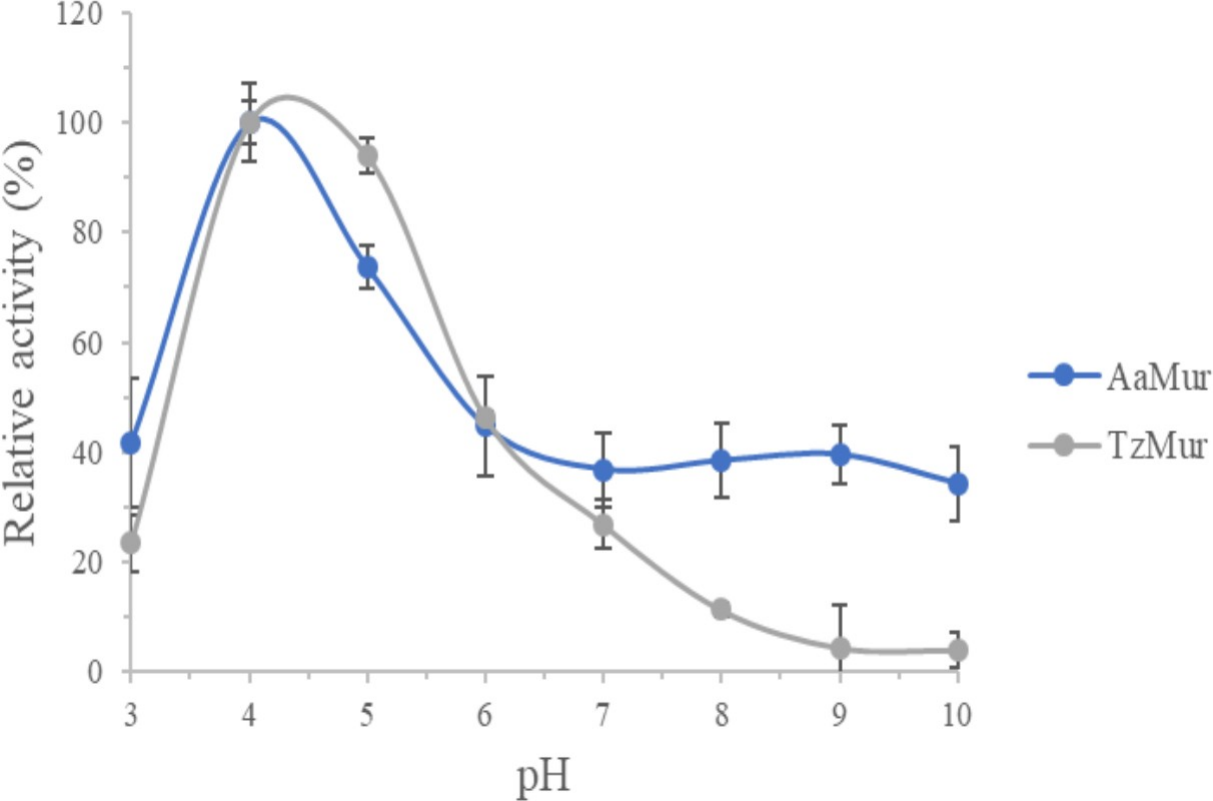
Activity profiles (activity in % relative to maximum for each muramidase) for *Aa*Mur and *T*zMur determined with the reducing ends assay versus pH.

The pH optimum for *Aa*Mur and *Tz*Mur was determined using the OD-drop assay and the reducing ends assay, both with *M*. *lysodeikticus* peptidoglycan as substrate. For both muramidases the optimum was around pH 4, independent of assay. The pH-profiles of *Aa*Mur and *Tz*Mur using the reducing ends assay is shown in Fig 3.

The optimum is at pH 4.0, but the activity profiles are quite broad, especially for *Aa*Mur. This is in the same range as for GH22 lysozymes from ruminants [33] and the stinkbird [13], while HEWL has its optimum at higher pH (6–7). However, the peptidoglycan molecule is a charged substrate with its own pI and since peptidoglycan varies dependent on species the resulting pH optimum will vary. The broad pH-profile favours *Aa*Mur over *Tz*Mur.

### 3.3. Crystal structures and sequence comparisons

**Fig 4:**
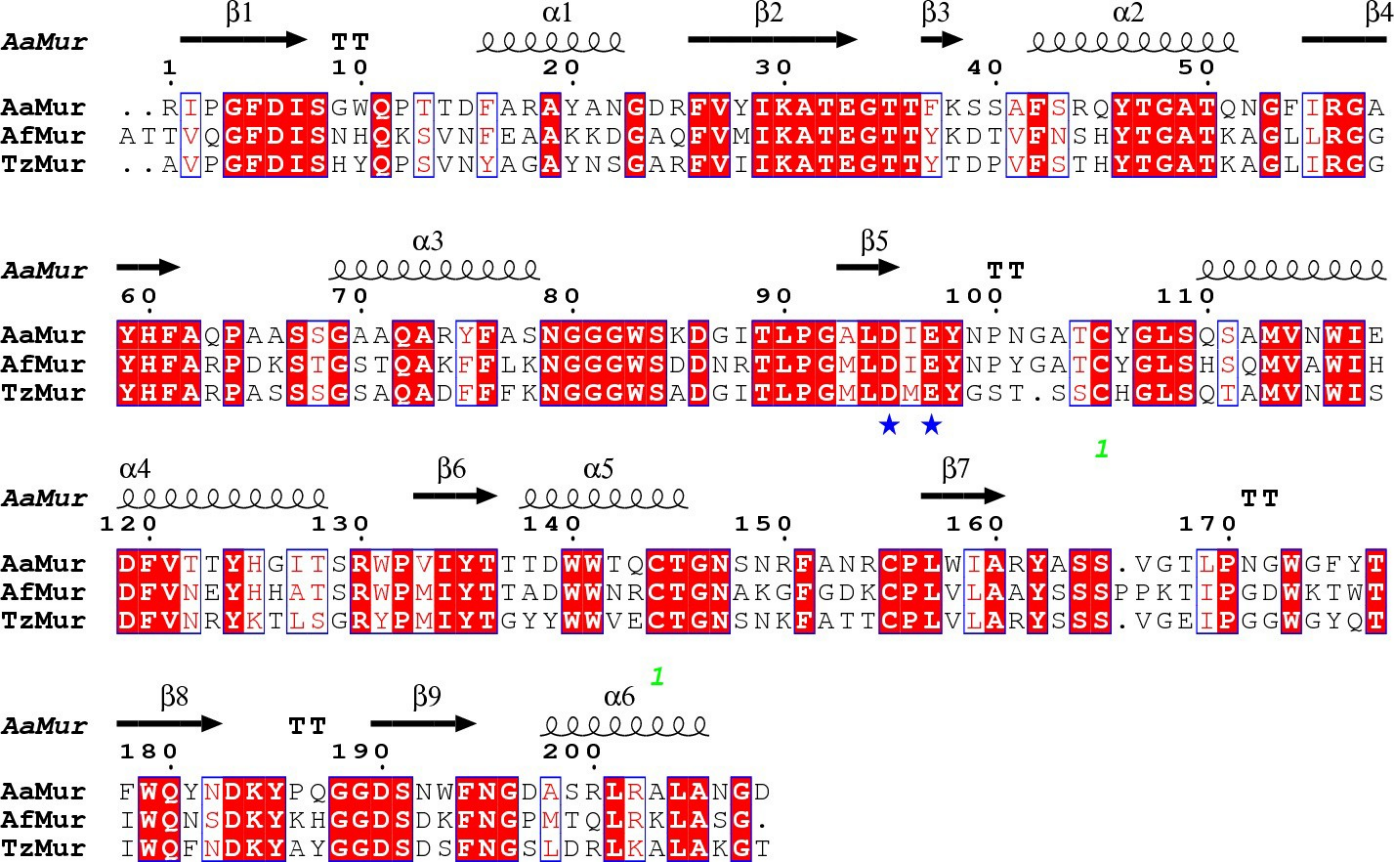
Alignment of the sequences of the three fungal enzymes for which structures are now available created with ESPript 3.0 [46]. Conserved residues are highlighted in red, conserved changes are in red font. The secondary structure of *Aa*Mur is shown above the top sequence, the catalytic residues (Asp95 and Glu97) are marked with a blue * and the position of the intramolecular disulfide bridge is indicated with an orange *. While both *Aa*Mur and *Tz*Mur have putative N-glycosylation sites (NRC for *Aa*Mur and NGS for *Tz*Mur), none of them were glycosylated at these sites in the structures. The pI of both *Aa*Mur and *Tz*Mur is 8.4.

The sequences of the three fungal muramidases with known structures (*Aa*Mur, *Tz*Mur and the previously published enzyme from *A*. *fumigatus* (*Af*Mur)) are between 58 and 63% identical, with the two catalytic residues (Asp95 and Glu97) located in the region corresponding to the first half of the (βα)_5_ (β)_3_ -barrel (discussed below). An alignment of the three sequences is shown in Fig 4.

#### 3.3.1. Acremonium alcalophilum

**Fig 5:**
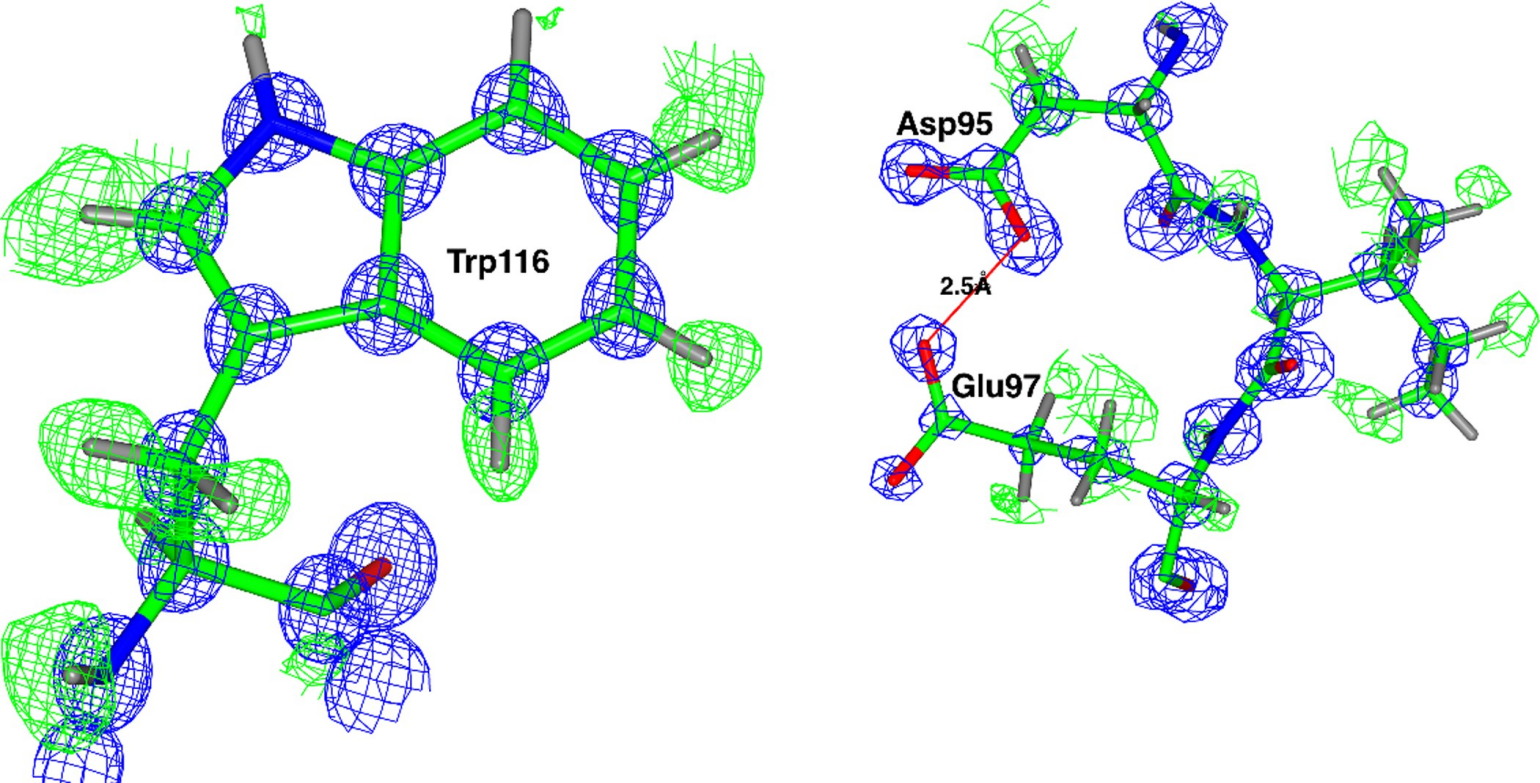
The electron density of the *Aa*Mur structure. With the REFMAC maximum likelihood weighted map contoured at 3 σ shown in blue, with the difference map contoured at 1.5 σ in green (positive) and red (negative), using phases calculated from the final model without the riding H-atoms. (a) The density for residue Trp116 and (b) for residues 95–97 including the Asp97 and Glu97 residues important for catalysis. A strong H-bond is shown between the catalytic acid Asp95 and its conserved supporting residue Glu97. The high quality of the structure at 0.78Å is evident from the weighted map and in the difference map there are positive density peaks for a good number of the hydrogen atoms.

For *AaMur*, there was a single molecule in the asymmetric unit and the whole chain was modelled, with only the terminal residues Arg1 and Asp208 being rather poorly defined. The catalytic residues in the conserved GH25 DXE motif are Asp95 and Glu97. The high resolution of the structure is exemplified by the density shown in Fig 5. The density is typical for such a high-resolution structure showing essentially resolved carbon, oxygen and nitrogen atoms, with many of the hydrogen atoms seen in the difference density.

#### 3.3.2. Trichobolus zukalii

For *TzMur*, there were two independent protein molecules in the asymmetric unit, and the catalytic residues are Asp95 and Glu97. There are sulphate molecules from the crystallisation medium–one in subunit A and two in B. There are four glycerol molecules bound in each chain, introduced when adding the cryoprotectant. Three of these (numbered 301–303) lie in or close to the substrate-binding site, with the fourth one at the opposite side of the molecule. There is some residual difference density in the active site not good enough to be described as a ligand, the situation being complicated because the final crystals were obtained from the re-dissolved drops collected from other trays due to shortage of material. This density is described as water.

**Fig 6:**
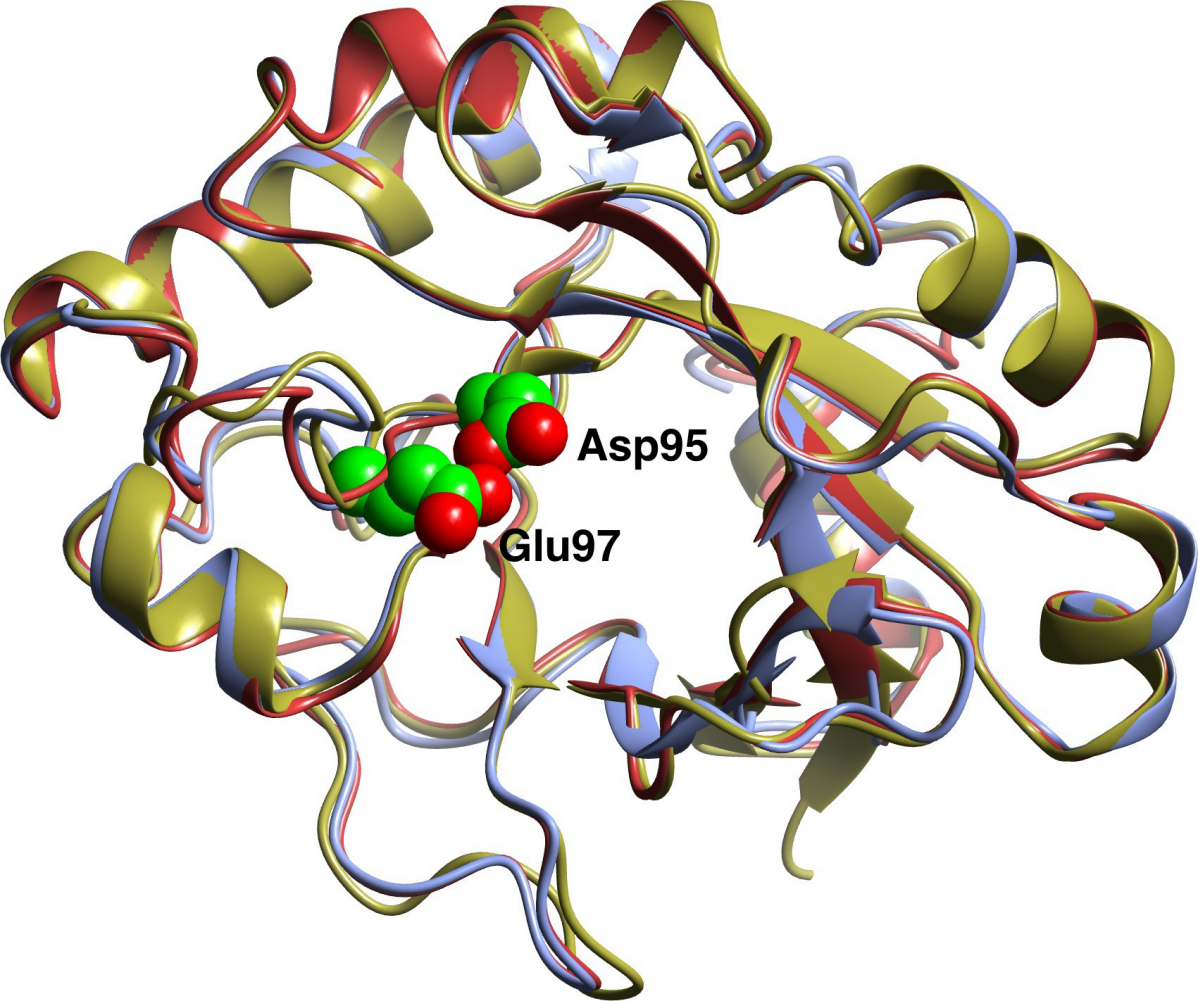
Ribbon diagram showing the superimposed folds of GH25 muramidases. *AaMur* is in ice blue, *TzMur* (Chain A) in brown) and *Af*Mur (2X8R, Chain A) in gold. The two catalytic residues for *Aa*Mur are shown as spheres–these correspond to Asp105 and Glu107 in 2X8R. The structures were superposed using the SSM option in CCP4mg.

Both proteins have the expected GH25-specific (βα)_5_ (β)_3_ fold with the active DXE motif located in the N-terminal half of the barrel (Fig 6). As mentioned before, this distorted, GH25—specific βα fold was first described for the structure of “Cellosyl”–a muramidase from *Streptomyces coelicolor*. where the last three β-strands are connected by loops without any helices, and the final β8 strand is in an unusual antiparallel orientation. The first half of the barrel, where the active site residues are located, is a classical barrel which is consistent with the idea that the initial ancient fold was a half-barrel [47] which became an eight stranded barrel by gene duplication, with more sequence conservation in the half that contains the catalytic residue and less sequence (and as seen here structure) conservation in the other half which is responsible for binding specific targets [48]. It is proposed that, as previously suggested for other GH25 muramidases, that Asp95 acts as an acid donating a proton to the glycosidic oxygen of the substrate, with the N-acetyl group of the substrate rather than a second protein carboxylate acting as the enzymatic nucleophile to stabilise the oxocarbenium ion-like transition state. This mechanism is also found in inter alia the chitobiases from family GH20 such as that from *Serratia marcescens* [49].

### 3.4. Comparison to known GH25 family structures–active site

**Fig 7:**
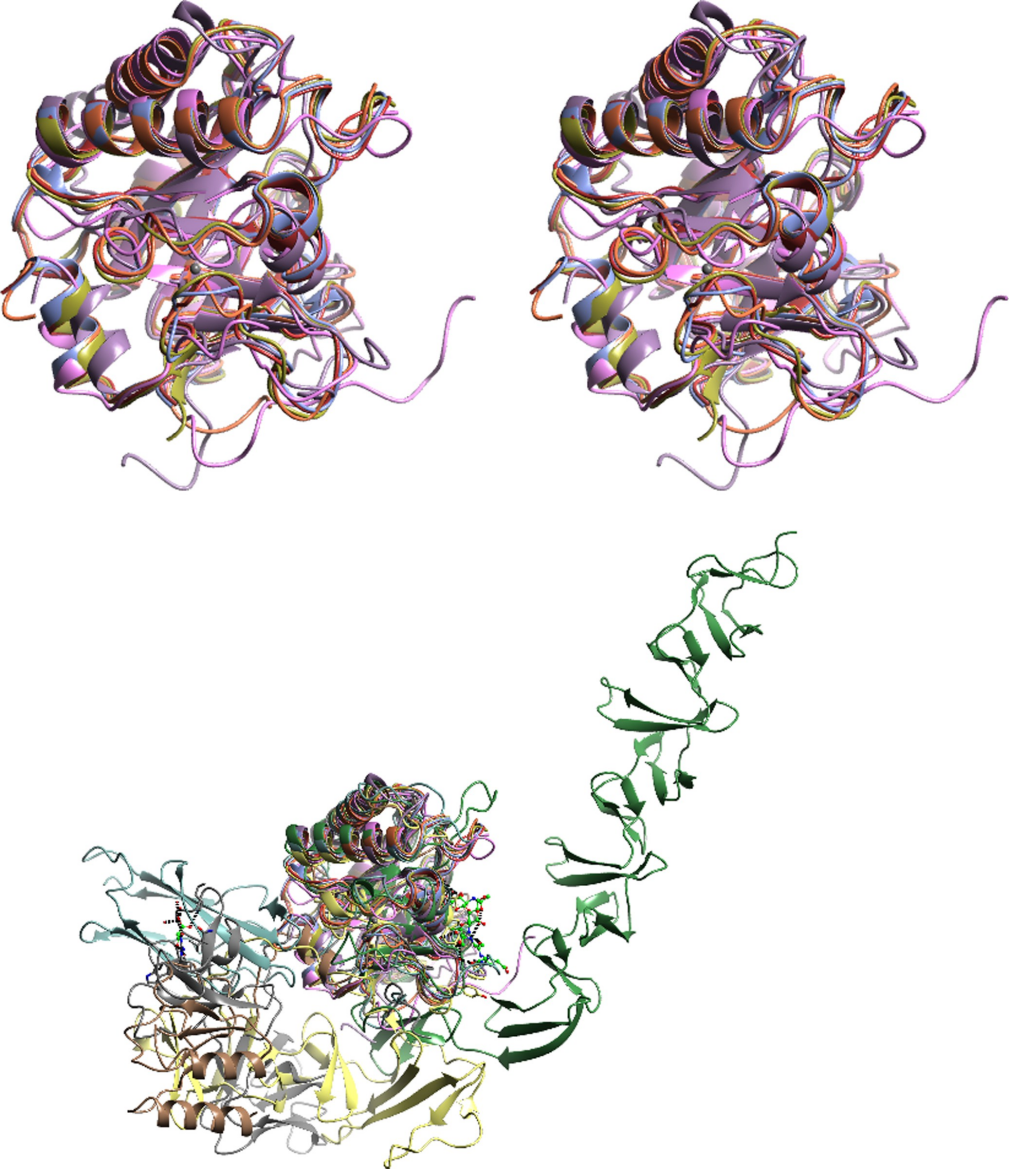
Structure comparisons. (a) Stereo view of a superposition of a representative set of GH25 enzymes (*Aa*Mur, *Tz*Mur and PDB codes 2X8R, 1JFX, 2WAG and 4JZ5) with a single catalytic domain. The folds are seen to be highly conserved. (b) Superposition of the same enzymes with a set containing additional domains (PDB codes: 4KRT, 5JIP, 5A6S, 2IXU and 2WWD).

As stated in the introduction, there are several GH25 structures already in the PDB–a representative set composed of a single catalytic domain is superposed in Fig 7A. Several GH25 family members have the catalytic domain as part of a much larger structure, Fig 7B. Details of these structures are available from the Cazy database (http://www.cazy.org/GH25.html) which currently reports structures for six bacterial, one fungal (*Aspergillus fumigatus*) and three viral (phage) GH25 enzymes. Structure comparisons for the structures reported here not surprisingly show the highest similarity to the *Aspergillus fumigatus* enzyme. The structures for these three fungal GH25s are highly conserved, with the rms difference compared to *AaMur* being 0.57 Å over 207 equivalent Cα atoms for *Tz*Mur and 1.07 Å over 204 Cα atoms for the *Aspergillus fumigatus* enzyme.

Soaking experiments with the aim of getting a ligand complex had previously been made in the course of the structure solution of *Af*Mur [30]. The first attempt involved soaking a chitotriothiazoline dithioamide inhibitor into both crystal forms of the wild type enzyme. This proved unsuccessful, data were collected but no ligand bound. The next attempt used inactive mutants of *Af*Mur (E100A and D98A) which were soaked with peptidoglycan analogues. In both experiments, crystals were obtained and data collected, but no ligand was seen in the resulting structures. In the present project we tried with supernatants (the soluble part) of peptidoglycan (which is insoluble) treated with *Aa*Mur, i.e. a product solution. The peptidoglycans used in these experiments were from two different *Lactobacilli* where *Aa*Mur has high activity (as seen with a reducing sugar assay). A few microliters were added to a crystal in mother liqueur. The hydrolysed peptidoglycan was in a ca. 35 mM citrate/120 mM phosphate buffer pH 6.0. Data were collected from the crystals, but no ligand could be seen in the density maps. The reason why no ligand binding was observed remains unclear: as all these experiments were unsuccessful, the details are not included here.

**Fig 8:**
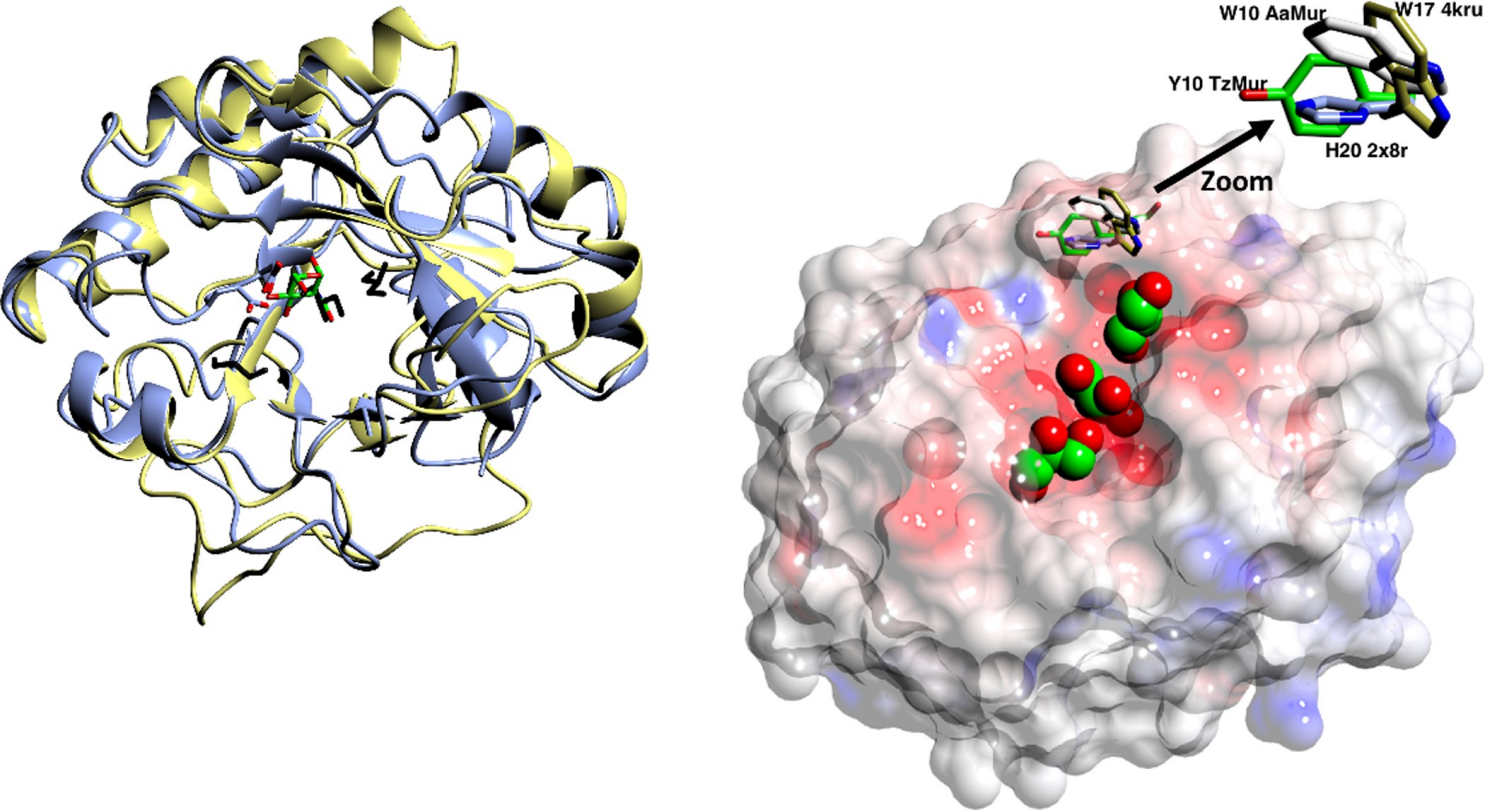
(a) Superposition of *Tz*Mur (ice blue) on 4KRU (yellow), showing one of glycerols (black) superimposing on part of NDG (green) bound in the catalytic domain of the endolysin from *Clostridium perfringens* phage phiSM101 (4KRU), both in the vicinity of active site D and E. The other two glycerols most likely indicate the positions where peptidoglycan would be in a complex. (b) Surface representation of *Tz*Mur with the binding site indicated with glycerol molecules. The aromatic residues corresponding to Tyr10 of *Tz*Mur in superimposed structures of *Aa*Mur, 2X8R and 4KRU all have different conformations, with flexibility at this location potentially facilitating optimal positioning of peptidoglycan for processing. The aromatic residues are shown as cylinders, green for *Tz*Mur, yellow for *Aa*Mur, ice blue for 2X8R and coral for 4KRU. Glycerols from *Tz*Mur are shown as spheres, they indicate the position of binding pocket.

**Fig 9:**
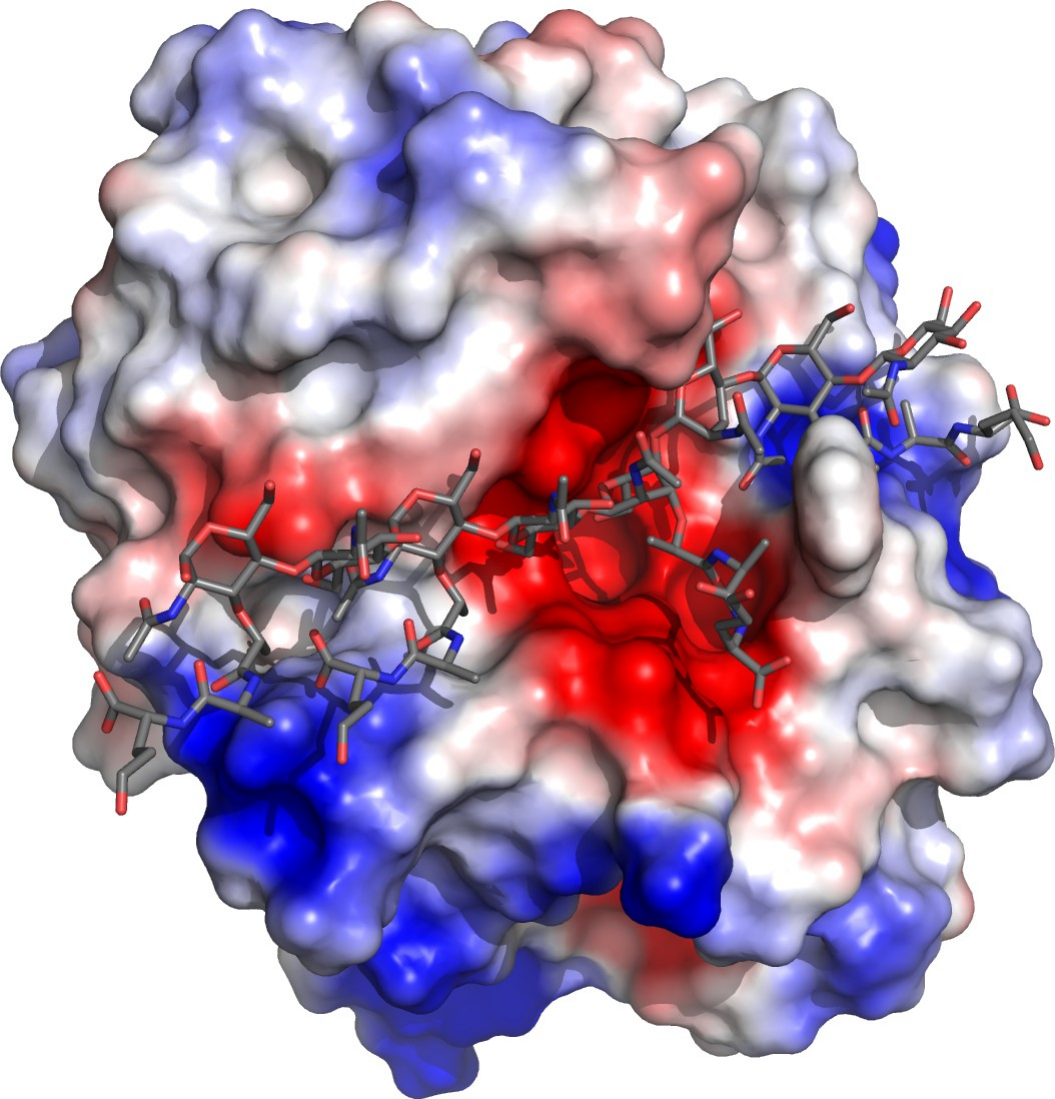
Model of ligand binding by AaMur. The surface of *Aa*Mur is shown. The ligand model was created by superposition of the *Aa*Mur structure and the 2WWD template to position the first sugar of the substrate. Three additional sugars were then placed using Pymol with the dihedrals adjusted to remove clashes as far as possible, while maintaining the coordinates of the substrate fragment resolved in 2WWD.

Three of the GH25 structures have ligands bound, and, while we have not succeeded in obtaining a ligand complex, the structure of *Tz*Mur has four glycerol molecules from the cryoprotectant, three of which are in the ligand binding site. One glycerol superimposes quite well on part of the NDG (2-(acetylamino)-2-deoxy-a-d-glucopyranose) ligand from the catalytic domain of the endolysin from *Clostridium perfringens* phage phiSM101 (PDB: 4KRU), both are close to the catalytic residues D95 and E97 (D101 and E103 in 4KRU) Fig 8A. While most of the surrounding side chains from the binding sites superimpose very well, Tyr10 of *Tz*Mur and the corresponding aromatic residues from the other superimposed structures all have different conformations, indicating a high flexibility of the entrance to the binding site, which might facilitate optimal positioning of peptidoglycan for processing. The importance of these residues for target recognition had been pointed out for Cpl-1 [50] and endolysin from phage phiSM101 [24], where they were in different conformations depending on whether or not the active site contained a ligand (Fig 8B). While the longer ligands from *Streptococcus* phage Cp-1 do not enter the binding pocket, another of the glycerols lies very close to the O6 of the NAGs seen in 2J8G and 2IXU, indicating a potential location of another part of peptidoglycan in *Tz*Mur and most probably other fungal muramidases. We finally placed a ligand into the binding cleft based on the structure of the modular autolysin LytC from *Streptococcus pneumoniae* in complex with a pneumococcal peptidoglycan fragment PDB 2WWD, Fig 9. This was achieved by simply superimposing the two sets of coordinates using SSM [51] and did not involve further molecular modelling or docking. As expected, the ligand chain fits beautifully into the enzyme.

## 4. Conclusions

We have cloned, expressed and purified a large number of new fungal GH25 muramidases and deposited the sequences for these enzymes in public databases. In the screening of these new enzymes we used the classical “lysozyme” OD-drop assay with *M*. *lysodeikticus* cells. This assay was also the basis in the gastric stability test. Very little comparative activity data was found for GH25 muramidases. As mentioned before, out of more than 10,000 sequences only 31 are listed as “characterized” in the CAZy-database. We found only four papers on GH25 enzymes that use variations of the OD-drop assay in their characterization [27,52–54]. We have also employed the reducing ends assay to monitor the solubilization of peptidoglycan by the muramidases. The assay can be performed in microtiter plates and the conditions (e.g. muramidase and substrate concentration and pH) can be varied. We believe this is the first time this method has been used to study GH25 muramidase and also on peptidoglycan in general.

The screening of muramidases from 47 fungi led to the selection of *Aa*Mur, the enzyme from *Acremonium alcalophilum* JCM 736, as that which had the optimal properties (gastric stable, moderate activity on peptidoglycan from two bacteria and good thermostability) and has resulted in a successful commercial product (Balancius^TM^ from DSM nutritional products, Kaiseraugst, Switzerland) that gives improved utilization of feed ingredients [16,17,19]. The crystal structures of *Aa*Mur and its close homologue *Tz*Mur have been determined thus increasing the number of structurally characterised fungal GH25 muramidases from one to three; the structures of fungal muramidases are compared to other GH25 family members. The new information presented here is important for future improvement of the role of these muramidases as feed additives, as well as for other potential applications.

While it is possible to relate features of the structure to the catalytic mechanism and substrate specificity, it remains challenging to directly relate structural features to such global properties as gastric stable, moderate activity on bacterial peptidoglycan and good thermostability and to predict with confidence how these properties can be enhanced. This would be a requirement of intelligent structure-based design of an enzyme with such enhanced properties. An alternative approach would be to carry out site saturation mutagenesis on the *A*. *alcalophilum* enzyme to this end. This would require a simple and rapid, probably plate based, screening for the targeted properties. This could probably be achieved for the enzyme activity at application temperature and perhaps for the temperature stability itself. Selected variants could then be individually assayed for their gastric stability and more complex properties.

The patent application “Polypeptides having lysozyme activity, polynucleotides encoding same and uses and compositions thereof” (WO 2018/113745) was based on the work described in this paper.

## Supporting information

S1 Text Cloning, expression, purification and activity determination. (DOCX)

S1 File (PDF)

S2 File (PDF)

## Abbreviations

CAZy: Carbohydrate-Active enZYmes Database
GH: glycoside hydrolase
HEWL: HEWL: hen egg white lysozyme
CAZy: Carbohydrate-Active enZYmes Database
*Aa*Mur: The muramidase from *Acremonium alcalophilum* JCM 736
*Tz*Mur: The muramidase from *Trichobolus zukalii*
NAG: *N*-acetylglucosamine
NAM: *N*-acetylmuramic acid
