# Supplementary material for: Fungal GH25 muramidases: New family members with applications in animal nutrition and a crystal structure at 0.78Å resolution
Source: PLoS One. 2021 Mar 12;16(3):e0248190. doi: 10.1371/journal.pone.0248190 (PMC7954357; doi:10.1371/journal.pone.0248190)
Supplement: S1 Text — (DOCX) [file pone.0248190.s001.docx]

# Supporting information

### Cloning and expression of AaMur and TzMur

*Aa*Mur was cloned and expressed in *T. reesei* as previously described [1]. *Tz*Mur was cloned and expressed in *Aspergillus oryzae* in a similar way, using genomic DNA from the *Trichobolus zukalii* CBS720.69 strain purchased from CBS-KNAW Fungal Biodiversity Centre. The genomic DNA was isolated according to standard procedures (Patent [WO2018113745-A1](http://novo.patentorder.com/pdf/WO2018113745-A1)). The raw reads of *T. zukalii* were assembled using the program Idba [2]. The assembled sequences were analysed using standard bioinformatics methods for gene identification and function prediction. GeneMark-ES fungal version [3] was used for gene prediction. Blastall version 2.2.10 [4] (ftp://ftp.ncbi.nlm.nih.gov/blast/executables/release/2.2.10/) and HMMER version 2.1.1 (National Center for Biotechnology Information (NCBI), Bethesda, MD, USA) were used to predict function based on structural homology.

The entire open reading frame encoding the *Tz*Mur gene was amplified from genomic DNA of Strain *T. zukalii* CBS720.69 using the following primer pair:

KKSC0311-F 5’-ACACAACTGGGGATCCACCATGAAGCTCACCACTTTTATCACG

KKSC0311-R 5’-CTAGATCTCGAGAAGCTTTTAAGTCCCCTTGGCAAGCG

The fragments were then cloned into BamHI and HindIII digested pDau109 (WO 2005/042735) using an IN-FUSION™ Cloning Kit. Protoplasts of *A. oryzae* MT3568 were prepared and used for transformation of the plasmid according to WO 95/002043. Transformants were spore-purified twice before fermentation in shake flasks. Analysis of the culture fluid from the fermentations by SDS–PAGE confirmed the presence of a protein band migrating at the expected rate for the molecular size (22 kDa).

## Purification of AaMur

Purification of the GH25s was carried out by standard techniques, typically involving cation or anion exchange chromatography (dependent on the pI of the target protein). Details are here given for *AaMur*, but *TzMur* was purified with the same procedures. The fermentation supernatant (from *T. reesei* or *A. oryzae*) was filtered through a Fast PES Bottle top filter with a 0.22 μm cut-off, and the pH was adjusted to 4.5 with 10% acetic acid. After the pH-adjustment the solution became slightly cloudy and was clarified by filtration through a Fast PES Bottle top filter with a 0.22 μm cut-off. Following this pre-treatment approximately 650 ml batches of the muramidase-containing solution were purified by cation-exchange chromatography on SP Sepharose, of an approximately 50 ml volume packed in a XK26 column, using 50 mM Na-acetate pH 4.5 as buffer A, and 50 mM Na-acetate plus 1 M NaCl pH 4.5 as buffer B. *Aa*Mur eluted from the column at app. 0.3 M NaCl and fractions were pooled based on the chromatogram (absorption at 280 and 254 nm) and SDS-PAGE analysis. The pooled fractions were buffer-exchanged into 50 mM Na-acetate, pH 5.5 and concentrated using Amicon spin filters with a 10 kDa cut-off.

The molecular weight, as estimated from SDS-PAGE, was approximately 22 kDa and the purity of both was > 95%. Intact molecule mass spectrometry confirmed the expected amino acid sequence (for *Aa*Mur, calculated: 23030.0 g/mol, observed: 23029.7 g/mol). This also shows that *Aa*Mur is not glycosylated in the *T. reesei* host (same for *Tz*Mur).

## Determination of muramidase activity against *Lactobacillus johnsonii.*

### Cultivation of Lactobacillus johnsonii

*Materials*

- MRS broth, product number BD 288130, pH 6.3-6.7.
- MRS agar plates, BD 288130; Agar Oxoid LP0011; pH 6.3-6.7.
- 0.9% NaCl, Merck 106404, Cas no. 7647145
- Jars, supplier Merck 116387, Anaerocult anaerobic jar 2.5 L
- Anaerogen 2,5L, ThermoScientific, catalogue no. AN0025A
- *Lactobacillus johnsonii,* DSM10533

*L. johnsonii* was streaked from freeze stock to MRS agar plate and incubated under anaerobic conditions for 2 days, anaerobic jar with Anaerogen 2.5 L, 30°C. Some colonies were inoculated into 500 mL MRS broth in a 500 mL blue cap bottle and placed in an anaerobic jar with Anaerogen 2.5 L for 72 hours at 30°C.

The culture was spun down (6000 rpm, 10 minutes) and the supernatant was poured off before another round of centrifugation was performed. The pellet was washed in 100 mL 0.9% NaCl and the suspension was mixed well and centrifuged at 6000 rpm for 10 minutes. The supernatant was poured off and the washing procedure in 0,9% NaCl was repeated to a total of three washes. Approximately 40 mL 0,9% NaCl was added to the pellet and the solution was transfered to a 50 mL falcon tube. The solution was centrifuged at 6000 rpm for 10 minutes and the supernatant was poured off. The pellet was stored at -18°C until the extraction of the peptidoglycan was conducted.

### Peptidoglycan extraction

*Materials*

- Protease from Streptomyces griseus, Sigma-Aldrich P5147, CAS 9036-06-0
- PBS pH 7.3:
- NaCl: 8 g, Sigma-Aldrich 31434, CAS 7647-14-5
- KCl: 0.2 g, Sigma-Aldrich P9333, CAS 7447-40-7
- KH_2_PO_4_: 0.24 g, Sigma-Aldrich P5655, CAS 7778-77-0
- Na_2_HPO_4_. 2 H_2_O: 1.44 g, Sigma-Aldrich 30412, CAS 10028-24-7
- Add Milli-Q water to 1000 mL
- 1% Triton-X 100 solution:
- 1 mL Triton X100, Sigma-Aldrich X100, CAS 9002-93-1
- Add Milli Q water to 100 mL
- 500 mM sodium carbonate buffer, pH 9.3:
- 500 mM sodium carbonate is made from 21 g Na_2_CO_3_ (Sigma-Aldrich S7795, CAS 497-19-8) in 500 mL MQ water
- 500 mM sodium bicarbonate is made from 72 g NaHCO_3_ (Sigma-Aldrich S6014, CAS 144-55-8) in 500 mL MW water
- The pH 9.3 buffer is made from 320 mL NaHCO_3_ and 80 mL Na_2_CO_3_ and adjusting pH with HCl
- Phenol solution with 10 mM Tris HCl, pH 8.0, 1 mM EDTA, Sigma-Aldrich P4557, CAS 108-95-2
- Acetone, Sigma-Aldrich 32201-M, CAS 67-64-1
- Ethanol, 96%, CCS Healthcare 1680643, CAS 64-17-5

*L. johnsonii* cell material was freeze dried. The freeze-dried material (525 mg) was suspended in PBS (40 mL) in a 50 mL Falcon tube. The suspension was shaken for 2 h @ 700 rpm in a thermoshaker at room temperature. Streptomyces griseus protease (55 mg) was then added and the suspension was incubated 6 h @ 37 C in the thermoshaker. It was then centrifuged 20 min @ 1900 g at room temperature, and the supernatant was decanted. The pellet was re-suspended in 1% Triton X-100 (40 mL) and shaken overnight @ 37 C. After another centrifugation and decantation, the pellet was re-suspended in PBS (40 mL) and protease (55 mg) added again. The suspension was again incubated 6h @ 37 C, centrifuged and decanted. The pellet was re-suspended in PBS (40 mL) and shaken overnight @ 37 C. This washing procedure was repeated once more with PBS (40 mL, 30 min agitation), then with 50% ethanol/water (40 mL, 30 min agitation). The pellet was then split into two Falcon tubes. To each tube was added phenol solution (15 mL) pre-heated to 40 C. The suspensions were shaken 10 min @ 40 C, and then added 96% ethanol (25 mL to each tube), centrifuged and decanted. The pellets were further washed with acetone (40 mL in each tube) and 96% ethanol (40 mL in each tube), before being freeze dried. Combining the pellets from the two tubes yielded 80 mg purified peptidoglycan as a white powder.

1. Lichtenberg, J.; Perez Calvo, E.; Madsen, K.; Ostergaard Lund, T.; Kramer Birkved, F.; van Cauwenberghe, S.; Mourier, M.; Wulf-Andersen, L.; Jansman, A. J. M.; Lopez-Ulibarri, R., Safety evaluation of a novel muramidase for feed application. *Regul Toxicol Pharmacol* **2017,** 89, 57-69.

2. Peng, Y.; Leung, H. C. M.; Yiu, S. M.; Chin, F. Y. L., IDBA - A Practical Iterative de Bruijn Graph De Novo Assembler. *Lect N Bioinformat* **2010,** 6044, 426-440.

3. Ter-Hovhannisyan, V.; Lomsadze, A.; Chernoff, Y. O.; Borodovsky, M., Gene prediction in novel fungal genomes using an ab initio algorithm with unsupervised training. *Genome Res* **2008,** 18, (12), 1979-90.

4. Altschul, S. F.; Gish, W.; Miller, W.; Myers, E. W.; Lipman, D. J., Basic local alignment search tool. *J Mol Biol* **1990,** 215, (3), 403-10.
