# Supplementary material for: Fungal GH25 muramidases: New family members with applications in animal nutrition and a crystal structure at 0.78Å resolution
Source: PLoS One. 2021 Mar 12;16(3):e0248190. doi: 10.1371/journal.pone.0248190 (PMC7954357; doi:10.1371/journal.pone.0248190)
Supplement: S1 File — (PDF) [file pone.0248190.s002.pdf]

Full wwPDB X-ray Structure Validation Report ⓘ

Jul 3, 2020 – 10:26 AM BST

PDB ID	:	6ZM8
Title	:	Structure of muramidase from Acremonium alcalophilum
Deposited on	:	2020-07-01
Resolution	:	0.78 Å(reported)

This is a Full wwPDB X-ray Structure Validation Report.

This report is produced by the wwPDB biocuration pipeline after annotation of the structure.

We welcome your comments at *validation@mail.wwpdb.org*
A user guide is available at
https://www.wwpdb.org/validation/2017/XrayValidationReportHelp
with specific help available everywhere you see the ⓘ symbol.

CONFIDENTIAL VALIDATION REPORT

The following versions of software and data (see references ⓘ) were used in the production of this report:

MolProbity	:	4.02b-467
Xtriage (Phenix)	:	1.13
EDS	:	2.13.dev1
Percentile statistics	:	20191225.v01 (using entries in the PDB archive December 25th 2019)
Refmac	:	5.8.0158
CCP4	:	7.0.044 (Gargrove)
Ideal geometry (proteins)	:	Engh & Huber (2001)
Ideal geometry (DNA, RNA)	:	Parkinson et al. (1996)
Validation Pipeline (wwPDB-VP)	:	2.13.dev1

1 Overall quality at a glance (i)

The following experimental techniques were used to determine the structure:
X-RAY DIFFRACTION

The reported resolution of this entry is 0.78 Å.

Percentile scores (ranging between 0-100) for global validation metrics of the entry are shown in the following graphic. The table shows the number of entries on which the scores are based.

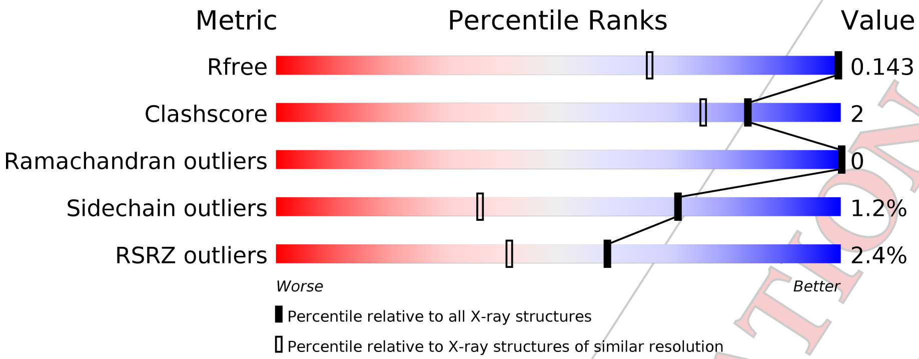

Metric	Whole archive (#Entries)	Similar resolution (#Entries, resolution range(Å))
R_{free}	130704	1080 (1.04-0.54)
Clashscore	141614	1154 (1.04-0.54)
Ramachandran outliers	138981	1072 (1.04-0.54)
Sidechain outliers	138945	1073 (1.04-0.54)
RSRZ outliers	127900	1045 (1.04-0.56)

The table below summarises the geometric issues observed across the polymeric chains and their fit to the electron density. The red, orange, yellow and green segments on the lower bar indicate the fraction of residues that contain outliers for >=3, 2, 1 and 0 types of geometric quality criteria respectively. A grey segment represents the fraction of residues that are not modelled. The numeric value for each fraction is indicated below the corresponding segment, with a dot representing fractions <=5% The upper red bar (where present) indicates the fraction of residues that have poor fit to the electron density. The numeric value is given above the bar.

Mol	Chain	Length	Quality of chain
1	A	208	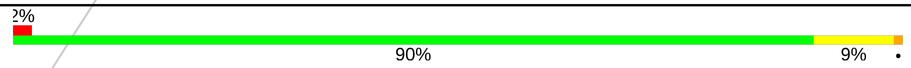

CONFIDENTIAL VALIDATION REPORT

2 Entry composition (i)

There are 2 unique types of molecules in this entry. The entry contains 3666 atoms, of which 1585 are hydrogens and 0 are deuteriums.

In the tables below, the ZeroOcc column contains the number of atoms modelled with zero occupancy, the AltConf column contains the number of residues with at least one atom in alternate conformation and the Trace column contains the number of residues modelled with at most 2 atoms.

- Molecule 1 is a protein called muramidase.

Mol	Chain	Residues	Atoms						ZeroOcc	AltConf	Trace
			Total	C	H	N	O	S			
1	A	208	3310	1093	1585	300	328	4	110	12	0

- Molecule 2 is water.

Mol	Chain	Residues	Atoms		ZeroOcc	AltConf
			Total	O		
2	A	356	356	356	0	0

CONFIDENTIAL VALIDATION REPORT

3 Residue-property plots (i)

These plots are drawn for all protein, RNA, DNA and oligosaccharide chains in the entry. The first graphic for a chain summarises the proportions of the various outlier classes displayed in the second graphic. The second graphic shows the sequence view annotated by issues in geometry and electron density. Residues are color-coded according to the number of geometric quality criteria for which they contain at least one outlier: green = 0, yellow = 1, orange = 2 and red = 3 or more. A red dot above a residue indicates a poor fit to the electron density (RSRZ > 2). Stretches of 2 or more consecutive residues without any outlier are shown as a green connector. Residues present in the sample, but not in the model, are shown in grey.

- Molecule 1: muramidase

Chain A: 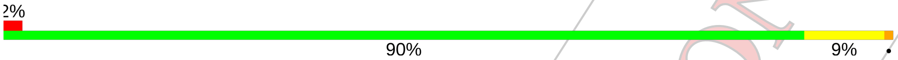

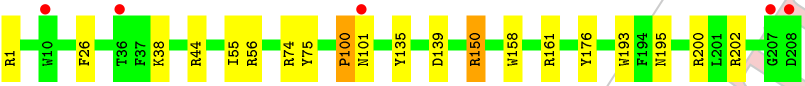

CONFIDENTIAL VALIDATION REPORT

4 Data and refinement statistics (i)

Property	Value	Source
Space group	P 1 21 1	Depositor
Cell constants<br>a, b, c, α, β, γ	34.09Å 77.27Å 35.73Å<br>90.00° 104.19° 90.00°	Depositor
Resolution (Å)	38.64 – 0.78<br>38.64 – 0.78	Depositor<br>EDS
% Data completeness<br>(in resolution range)	99.0 (38.64-0.78)<br>99.1 (38.64-0.78)	Depositor<br>EDS
R_{merge}	(Not available)	Depositor
R_{sym}	(Not available)	Depositor
$< I/\sigma(I) >$ [1]	1.34 (at 0.78Å)	Xtriage
Refinement program	REFMAC 5.8.0258	Depositor
R, R_{free}	0.122 , 0.134<br>0.131 , 0.143	Depositor<br>DCC
R_{free} test set	10021 reflections (4.99%)	wwPDB-VP
Wilson B-factor (Å^2)	6.7	Xtriage
Anisotropy	0.340	Xtriage
Bulk solvent k_{sol}(e/Å^3), B_{sol}(Å^2)	0.37 , 44.8	EDS
L-test for twinning[2]	$< \|L\| >$ = 0.50, $< L^2 >$ = 0.33	Xtriage
Estimated twinning fraction	0.019 for l,-k,h	Xtriage
F_o,F_c correlation	0.99	EDS
Total number of atoms	3666	wwPDB-VP
Average B, all atoms (Å^2)	10.0	wwPDB-VP

Xtriage's analysis on translational NCS is as follows: *The largest off-origin peak in the Patterson function is 8.71% of the height of the origin peak. No significant pseudotranslation is detected.*

[1] Intensities estimated from amplitudes.

[2] Theoretical values of $< |L| >$, $< L^2 >$ for acentric reflections are 0.5, 0.333 respectively for untwinned datasets, and 0.375, 0.2 for perfectly twinned datasets.

CONFIDENTIAL VALIDATION REPORT

5 Model quality (i)

5.1 Standard geometry (i)

The Z score for a bond length (or angle) is the number of standard deviations the observed value is removed from the expected value. A bond length (or angle) with $|Z| > 5$ is considered an outlier worth inspection. RMSZ is the root-mean-square of all Z scores of the bond lengths (or angles).

Mol	Chain	Bond lengths		Bond angles	
		RMSZ	#$\|Z\|$ >5	RMSZ	#$\|Z\|$ >5
1	A	0.69	2/1794 (0.1%)	0.95	8/2444 (0.3%)

Chiral center outliers are detected by calculating the chiral volume of a chiral center and verifying if the center is modelled as a planar moiety or with the opposite hand.A planarity outlier is detected by checking planarity of atoms in a peptide group, atoms in a mainchain group or atoms of a sidechain that are expected to be planar.

Mol	Chain	#Chirality outliers	#Planarity outliers
1	A	0	1

All (2) bond length outliers are listed below:

Mol	Chain	Res	Type	Atoms	Z	Observed(Å)	Ideal(Å)
1	A	100	PRO	CA-C	6.24	1.65	1.52
1	A	100	PRO	C-O	-6.10	1.11	1.23

All (8) bond angle outliers are listed below:

Mol	Chain	Res	Type	Atoms	Z	Observed(^{o})	Ideal(^{o})
1	A	150	ARG	NE-CZ-NH2	-9.68	115.46	120.30
1	A	161	ARG	NE-CZ-NH1	9.15	124.88	120.30
1	A	74	ARG	NE-CZ-NH1	-7.16	116.72	120.30
1	A	100	PRO	CA-C-O	-6.40	104.84	120.20
1	A	44	ARG	NE-CZ-NH1	-6.26	117.17	120.30
1	A	101	ASN	CA-CB-CG	-5.44	101.43	113.40
1	A	100	PRO	CA-C-N	5.08	128.38	117.20
1	A	100	PRO	N-CA-CB	5.00	109.30	103.30

There are no chirality outliers.

All (1) planarity outliers are listed below:

CONFIDENTIAL VALIDATION REPORT

Mol	Chain	Res	Type	Group
1	A	100	PRO	Mainchain

5.2 Too-close contacts (i)

In the following table, the Non-H and H(model) columns list the number of non-hydrogen atoms and hydrogen atoms in the chain respectively. The H(added) column lists the number of hydrogen atoms added and optimized by MolProbity. The Clashes column lists the number of clashes within the asymmetric unit, whereas Symm-Clashes lists symmetry related clashes.

Mol	Chain	Non-H	H(model)	H(added)	Clashes	Symm-Clashes
1	A	1725	1585	1579	8	1
2	A	356	0	0	2	2
All	All	2081	1585	1579	8	2

The all-atom clashscore is defined as the number of clashes found per 1000 atoms (including hydrogen atoms). The all-atom clashscore for this structure is 2.

All (8) close contacts within the same asymmetric unit are listed below, sorted by their clash magnitude.

Atom-1	Atom-2	Interatomic distance (Å)	Clash overlap (Å)
1:A:38[A]:LYS:HG3	1:A:75:TYR:OH	2.06	0.54
1:A:38[B]:LYS:HG3	1:A:75:TYR:OH	2.10	0.51
1:A:1:ARG:HG2	1:A:193:TRP:HB3	1.93	0.49
1:A:135:TYR:HA	1:A:158:TRP:O	2.13	0.49
1:A:176[B]:TYR:CE1	1:A:195:ASN:HB2	2.52	0.45
1:A:26:PHE:HA	1:A:55:ILE:O	2.17	0.44
1:A:200:ARG:HD2	2:A:535:HOH:O	2.16	0.44
1:A:202[A]:ARG:HD2	2:A:321:HOH:O	2.20	0.42

All (2) symmetry-related close contacts are listed below. The label for Atom-2 includes the symmetry operator and encoded unit-cell translations to be applied.

Atom-1	Atom-2	Interatomic distance (Å)	Clash overlap (Å)
1:A:139:ASP:OD1	2:A:323:HOH:O[1_455]	1.86	0.34
2:A:512:HOH:O	2:A:556:HOH:O[2_545]	1.96	0.24

CONFIDENTIAL VALIDATION REPORT

5.3 Torsion angles (i)

5.3.1 Protein backbone (i)

In the following table, the Percentiles column shows the percent Ramachandran outliers of the chain as a percentile score with respect to all X-ray entries followed by that with respect to entries of similar resolution.

The Analysed column shows the number of residues for which the backbone conformation was analysed, and the total number of residues.

Mol	Chain	Analysed	Favoured	Allowed	Outliers	Percentiles	
1	A	219/208 (105%)	214 (98%)	5 (2%)	0	100	100

There are no Ramachandran outliers to report.

5.3.2 Protein sidechains (i)

In the following table, the Percentiles column shows the percent sidechain outliers of the chain as a percentile score with respect to all X-ray entries followed by that with respect to entries of similar resolution.

The Analysed column shows the number of residues for which the sidechain conformation was analysed, and the total number of residues.

Mol	Chain	Analysed	Rotameric	Outliers	Percentiles	
1	A	174/161 (108%)	172 (99%)	2 (1%)	73	40

All (2) residues with a non-rotameric sidechain are listed below:

Mol	Chain	Res	Type
1	A	56	ARG
1	A	150	ARG

Some sidechains can be flipped to improve hydrogen bonding and reduce clashes. There are no such sidechains identified.

5.3.3 RNA (i)

There are no RNA molecules in this entry.

CONFIDENTIAL VALIDATION REPORT

5.4 Non-standard residues in protein, DNA, RNA chains (i)

There are no non-standard protein/DNA/RNA residues in this entry.

5.5 Carbohydrates (i)

There are no carbohydrates in this entry.

5.6 Ligand geometry (i)

There are no ligands in this entry.

5.7 Other polymers (i)

There are no such residues in this entry.

5.8 Polymer linkage issues (i)

There are no chain breaks in this entry.

CONFIDENTIAL VALIDATION REPORT

6 Fit of model and data (i)

6.1 Protein, DNA and RNA chains (i)

In the following table, the column labelled '#RSRZ> 2' contains the number (and percentage) of RSRZ outliers, followed by percent RSRZ outliers for the chain as percentile scores relative to all X-ray entries and entries of similar resolution. The OWAB column contains the minimum, median, 95th percentile and maximum values of the occupancy-weighted average B-factor per residue. The column labelled 'Q< 0.9' lists the number of (and percentage) of residues with an average occupancy less than 0.9.

Mol	Chain	Analysed	<RSRZ>	#RSRZ>2			OWAB(Å^2)	Q<0.9
1	A	208/208 (100%)	-0.53	5 (2%)	59	41	5, 7, 14, 78	0

All (5) RSRZ outliers are listed below:

Mol	Chain	Res	Type	RSRZ
1	A	101	ASN	4.7
1	A	207	GLY	4.7
1	A	36	THR	2.3
1	A	10	TRP	2.2
1	A	208	ASP	2.0

6.2 Non-standard residues in protein, DNA, RNA chains (i)

There are no non-standard protein/DNA/RNA residues in this entry.

6.3 Carbohydrates (i)

There are no carbohydrates in this entry.

6.4 Ligands (i)

There are no ligands in this entry.

6.5 Other polymers (i)

There are no such residues in this entry.

CONFIDENTIAL VALIDATION REPORT
